# Coronary Obstruction Following Transcatheter Aortic Valve Replacement With Supra-Annular Self-Expanding Valve: Review of Case Reports

**DOI:** 10.1016/j.jacadv.2025.102316

**Published:** 2025-10-25

**Authors:** Tomohiko Taniguchi, Aoi Omori, Toshiaki Toyota, Shinichi Shirai, Masaomi Hayashi, Hiroshi Takiguchi, Kenichi Ishizu, Hiroyuki Tabata, Kenji Ando, Yutaka Furukawa

**Affiliations:** a Department of Cardiovascular Medicine, Kobe City Medical Center General Hospital, Kobe, Japan; b Department of Cardiology, Kokura Memorial Hospital, Kitakyushu, Japan; c Department of Cardiovascular Medicine and Hypertension, Kagoshima University, Kagoshima, Japan

**Keywords:** aortic stenosis, coronary obstruction, transcatheter aortic valve replacement

Coronary obstruction following transcatheter aortic valve replacement (TAVR) in native aortic stenosis (AS) is rare (<1%) but life-threatening, with high in-hospital mortality.^1^^,^^2^ Coronary obstruction following TAVR can be stratified into acute (intraprocedural) or delayed, with the latter further subdivided into early (≤7 days) and late (>7 days). Acute coronary obstruction has been more frequently associated with balloon-expandable valves,^1^ whereas delayed coronary obstruction appears to be more common with self-expanding valves, such as CoreValve/Evolut (Medtronic).^2^ CoreValve/Evolut valves differ in waist diameter and frame geometry by size. However, there remains limited data on the specific relationship between valve size and the timing, mechanisms, and clinical presentation of coronary obstruction. This study aimed to systemically review published reports of coronary obstruction following TAVR with CoreValve/Evolut valves in native AS.

**What is the clinical question being addressed?**

What are the characteristics of coronary obstruction in patients undergoing transcatheter aortic valve replacement with CoreValve/Evolut for native aortic stenosis?

**What is the main finding?**

The 26-mm CoreValve/Evolut was most frequently implicated; obstruction mechanisms varied by timing, including leaflet displacement, narrow sinus of Valsalva, high implantation, sinus sequestration, and endothelialization.

## Methods

A systematic review of the literature on TAVR and coronary obstruction published from March 2007 (European market introduction) to January 2025 was conducted using PubMed, Ovid MEDLINE, and Cochrane Library in accordance with the Preferred Reporting Items for Systematic Reviews and Meta-Analyses 2020 guidelines. Inclusion criteria encompassed coronary obstruction involving the left main or ostial right coronary artery confirmed by angiography, surgery, or autopsy.^2^ Exclusion criteria included cases with balloon-expandable or non-CoreValve/Evolut valves, valve-in-valve procedures, embolic causes, prophylactic coronary protection or stenting, valve embolization during TAVR, and missing valve size information. Two reviewers (T. Taniguchi and A.O.) independently screened studies, with a third (T. Toyota) resolving discrepancies. The last literature search was completed on March 1, 2025. Baseline clinical and procedural data were extracted. High implantation was defined as <3 mm from the left coronary cusp in angiography or computed tomography, and a narrow sinus of Valsalva (SOV) was defined per Medtronic sizing thresholds. The review was registered with PROSPERO (CRD420251018679), and all statistical analyses were performed using JMP 18, with significance defined as *P* < 0.05.

## Results

Of 2,606 records screened, 33 publications describing 39 cases (mean age: 82.6 ± 6.4 years; 92% women) were included. The most commonly implicated valve was the 26-mm CoreValve/Evolut (32 cases), followed by the 29-mm (6 cases) and 23-mm (1 case), with no cases involving a 31-mm CoreValve or a 34-mm Evolut. Geographically, 10 cases were reported from the United States, 14 cases from Europe, and 15 cases from Asia. Among 24 patients with SOV data, 10 patients (42%) had an SOV diameter below the recommended threshold by Medtronic, and 21 patients (88%) had ≤3 mm of clearance between the implanted valve and the mean SOV. Low coronary height (<12 mm) was observed in 53%. The majority (82%) presented with acute coronary syndrome, and 33% required mechanical circulatory support. The left coronary artery was involved in 85%. Emergent intervention was needed in 82% of patients (percutaneous coronary intervention [PCI] 72%, surgery 28%), with 10% in-hospital mortality.

Among 19 patients with acute or early obstruction and 20 with late obstruction, leaflet displacement was the leading mechanism in acute or early obstruction (68% [N = 13/19]), whereas high implantation, sinus sequestration, and endothelialization were more common in late obstruction than in acute or late obstruction (80% vs 26%, *P* < 0.001; 70% vs 26%, *P* = 0.006; 85% vs 0%, *P* < 0.001, respectively). A narrow SOV and low coronary height (<12 mm) were observed in approximately half of the cases in both groups, although it was not significantly different between the 2 groups. PCI proved feasible in most cases; however, PCI failure was numerically higher in late obstruction (33% vs 13%, *P* = 0.20). Emergent surgical treatment was more frequently required in late obstruction (45% vs 11%, *P* = 0.02).

## Discussion

**Figure 1:**
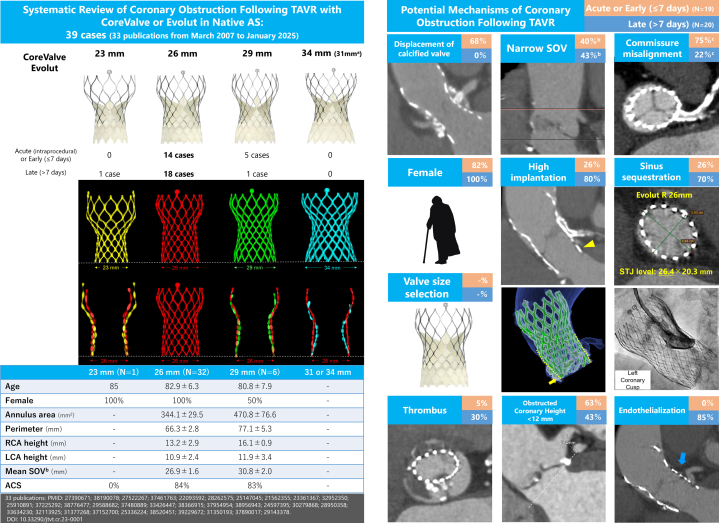
**CoreValve/Evolut Sizes and Factors Associated with Coronary Obstruction Following Transcatheter Aortic Valve Replacement in Native Aortic Stenosis** ^a^Maximum CoreValve size: 31 mm; Evolut up to 34 mm. ^b^Narrow SOV defined as <25 mm (23-mm valve), <27 mm (26-mm valve), and <29 mm (29-mm valve) per Medtronic IFU; SOV data available in 24/39 cases. ^c^Commissure alignment reported in 13/39 cases. All valve sizes were superimposed on the 26-mm valve (red), with bases aligned. The 26-mm valve showed the most cylindrical lower shape. High implantation, defined as <3 mm depth from the left coronary cusp (yellow arrows), allowed further expansion above the outer skirt (yellow dot lines). Aortography and CT revealed no left coronary flow due to sinus sequestration with endothelialization (blue arrow). The 26-mm Evolut R expanded elliptically (26.4 × 20.3 mm at STJ), contacting the aortic wall directly with minimal residual space between the valve and surrounding structures. ACS = acute coronary syndrome; AS = aortic stenosis; LCA = left coronary artery; RCA = right coronary artery; SOV = sinus of Valsalva; STJ = sinotubular junction; TAVR = transcatheter aortic valve replacement.

This is the first systematic review to identify the 26-mm CoreValve/Evolut as the valve most commonly associated with coronary obstruction, despite not being the most frequently implanted in Western populations. In the United States and Europe, the 29-mm valve is more commonly used,^3^ suggesting that structural features of the 26-mm valve may contribute inherently to obstruction risk. The 26-mm frame’s cylindrical lower design is likely to result in a smaller gap between the prosthetic valve and the SOV/sinotubular junction (STJ) after implantation, especially in patients with small aortic roots (Figure 1).

High implantation, observed in 63% (N = 20/32) of patients with 26-mm valves, allows greater expansion of the valve’s upper frame, further decreasing sinus space and promoting sinus sequestration or endothelial overgrowth in the late phase. Half of patients (N = 9/18) with available SOV data who experienced coronary obstruction with a 26-mm CoreValve/Evolut had a SOV diameter <27 mm, below the manufacturer’s recommended threshold, despite meeting the annulus perimeter criteria by Medtronic. In patients with narrow SOV and significant aortic valve calcification, the use of a downsized CoreValve/Evolut might increase the gap between the prosthetic valve and SOV, potentially preventing coronary obstruction without significant perivalvular leakage or severe patient-prosthetic mismatch.^4^ Delayed coronary obstruction was observed in 95% of cases (N = 19/20) more than 3 months post-TAVR, likely attributable to endothelialization or thrombus formation in the delayed setting. Endothelialization might contribute to the difficulty in valve snaring or PCI in such cases.

This study had several limitations. First, this descriptive study cannot robustly assess the incidence of coronary obstruction or draw strong conclusions about higher obstruction risk with 26-mm valves. Second, publication bias may overestimate procedural success. Third, incomplete computed tomography data limited the assessment of aortic valve calcification and the relationship between the implanted valve size and SOV diameter. Fourth, antithrombotic therapy details were unavailable for all, but coronary obstruction occurred under treatment in 7 cases.

## Conclusions

Coronary obstruction following TAVR with CoreValve/Evolut occurred most frequently with the 26-mm valve, despite not being the most commonly implanted size. The underlying mechanisms and optimal management differed according to the timing of coronary obstruction, underscoring the importance of individualized procedural planning. Further studies are essential to confirm these observations.

## Funding support and author disclosures

This work was supported by JSPS KAKENHI, Japan (Grant Number JP24K19020); Takeda Science Foundation, Japan; and Katakami Foundation for Clinical Research, Japan. Drs Taniguchi, Hayashi, Ishizu, and Tabata have received lecture fee from the Japanese affiliate of Edwards Lifesciences, Medtronic, and Abbott. All other authors have reported that they have no relationships relevant to the contents of this paper to disclose.
